# Characterizing an Optically Induced Sub-micrometer Gigahertz Acoustic Wave in a Silicon Thin Plate

**DOI:** 10.1021/acs.nanolett.2c03938

**Published:** 2023-03-21

**Authors:** Asuka Nakamura, Takahiro Shimojima, Kyoko Ishizaka

**Affiliations:** † RIKEN Center for Emergent Matter Science, Wako, Saitama 351-0198, Japan; ‡ Quantum-Phase Electronics Center and Department of Applied Physics, The University of Tokyo, Hongo, Tokyo 113-8656, Japan

**Keywords:** Ultrafast transmission electron microscopy, Nanoscale photoacoustic wave, Spatiotemporal Fourier-transform analysis, Finite-element method

## Abstract

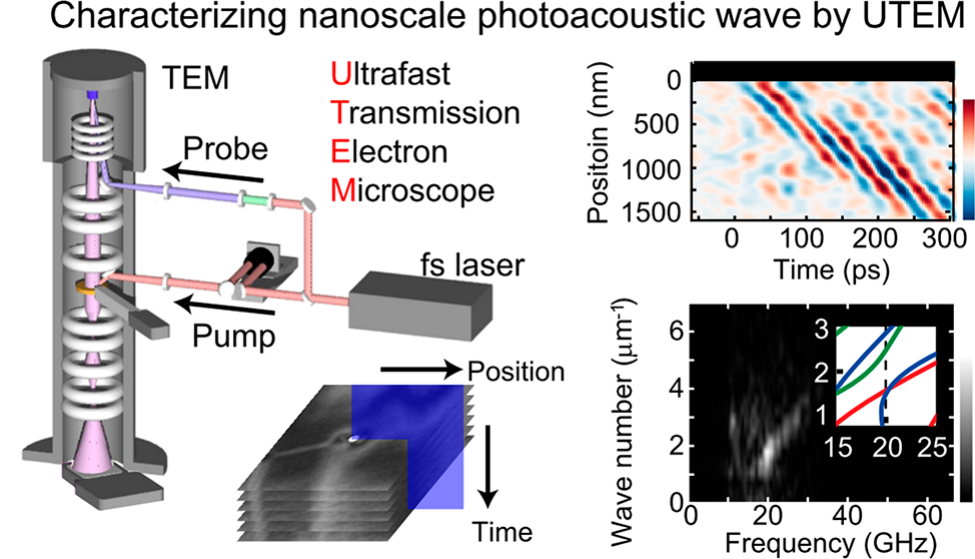

Optically induced GHz-THz guided acoustic waves have been intensively studied because of the potential to realize noninvasive and noncontact material inspection. Although the generation of photoinduced guided acoustic waves utilizing nanostructures, such as ultrathin plates, nanowires, and materials interfaces, is being established, experimental characterization of these acoustic waves in consideration of the finite size effect has been difficult due to the lack of experimental methods with nm × ps resolution. Here we experimentally observe the sub-micrometer guided acoustic waves in a nanofabricated ultrathin silicon plate by ultrafast transmission electron microscopy with nm × ps precision. We successfully characterize the excited guided acoustic wave in frequency-wavenumber space by applying Fourier-transformation analysis on the bright-field movie. These results suggest the great potential of ultrafast transmission electron microscopy to characterize the acoustic modes realized in various nanostructures.

Acoustic waves propagating along the surface of an elastic medium, i.e. guided waves, have been extensively studied since Lord Rayleigh predicted the existence of surface acoustic waves in 1885.^1^ Depending on the morphology of elastic media, including plates, wires, and semi-infinite surfaces, there are various types of guided wave modes.^2^ In general, guided waves decay more slowly with distance than bulk waves because of the confinement, which is preferable in terms of application. In particular, photoinduced GHz-THz acoustic waves are used as a tool for contactless and noninvasive material inspection due to the high sensitivity to defects in solids.^3,4^ As reported in the pioneering works in the 1980’s,^5,6^ photoexcitation of metals and semiconductors with ultrashort laser pulses can generate GHz-THz acoustic waves on picosecond time-scale at the materials’ surface through sudden lattice heating (thermoelastic effect) and/or deformation potential induced by photocarriers.^4,5,7,8^

Recent studies further utilize the acoustic properties in nanostructures such as ultrathin plates,^10^ nanowires,^11^ semiconductor superlattice,^9^ semiconductor interface,^12^ and phononic crystal.^13^ For example, when an ultrathin semiconductor plate is irradiated by pulsed light with penetration depth much larger than the sample thickness, the sample is photoexcited almost uniformly along the out-of-plane direction [Figure 1(a)]. At sample edges and/or defects [in the present case, a fabricated “hole” at the center as shown in Figures 1(a,b)], an in-plane guided wave is generated^14,15^ owing to the photoinduced stresses, which we refer to as a nanoplate wave in this paper. The wavelength of such a nanoplate wave is mainly determined by the sample thickness, *d*, and therefore is not affected by the diffraction limit of light used for photoexcitation.

**Figure 1:**
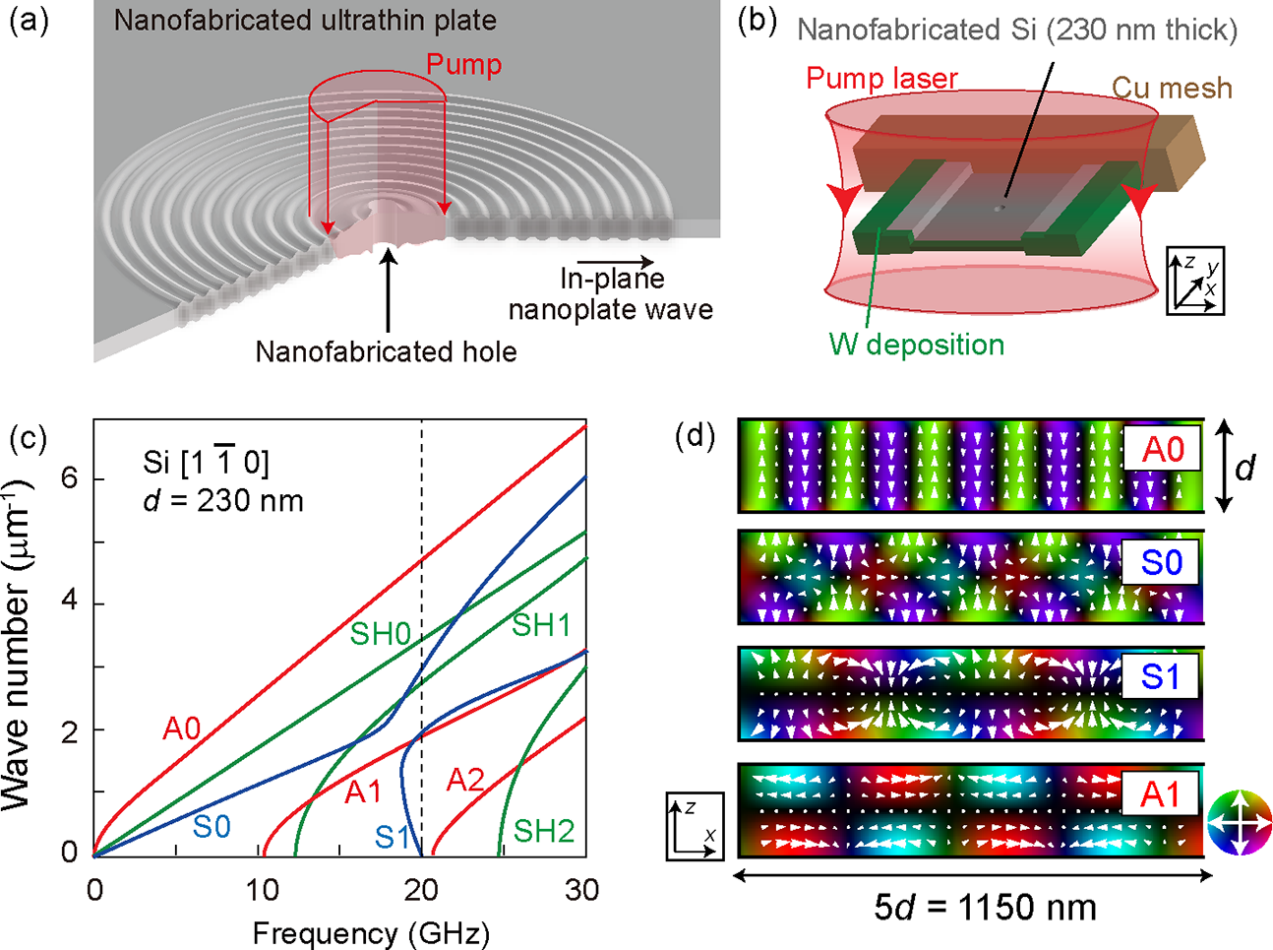
(a) Schematic of photoinduced nanoplate wave in an ultrathin plate. The sample is almost homogeneously excited along the out-of-plane direction, and nanoplate waves are generated at the edge of the central hole. (b) Schematic of the nanofabricated silicon sample that is used for the present measurements. 230-nm-thick silicon is attached to the copper mesh via tungsten deposition. At the center of the silicon thin plate, a hole with a 200 nm diameter is fabricated. (c) Calculated dispersion relation of a [11̅0] single crystalline silicon thin plate with a thickness of 230 nm. S0–S1 (A0–A2) represent symmetric (asymmetric) plate waves, exhibiting symmetric (asymmetric) atomic displacement along the thickness direction, as shown in part d. These dispersion curves are calculated by a partial wave method^2^ as described in Section 1 in the Supporting Information. (d) Atomic displacement of A0, S0, S1, and A1 plate waves in the *x-z* plane at 20 GHz frequency.

In contrast to efforts on generation of nanometric acoustic waves, experimental characterization of acoustic modes in nanostructures (including relatively simple nanoplate waves) have been very limited, mainly due to the lack of experimental methods with nm × ps resolution. In nanostructures, the boundary condition at the surfaces significantly modifies the nature of the acoustic waves.^2^Figures 1(c,d) depict the simulation of the frequency-wavenumber (*f*-*k*) dispersion curves and corresponding atomic displacements of nanoplate waves in a 230–nm-thick silicon [11̅0] thin plate^2,16^ (see Section 1 in the Supporting Information). In contrast to the simple bulk acoustic modes, namely one longitudinal and two transverse modes, there are multiple branches that exhibit singular behaviors with strong dispersions. Therefore, experimental evaluation of the dispersion curves and distribution function in *f*-*k* space is crucial to understand the guided waves in nanostructured systems. Although some works utilizing optical methods have been made and *f*-*k* dispersion is determined in the sub-GHz × μm^–1^ range,^17^ characterization of acoustic waves in higher *f* (>GHz) and large *k* (>μm^–1^) ranges has been yet challenging.

Recent development of ultrafast transmission electron microscopy opens a novel pathway to the direct observation of strain dynamics on nm × ps resolution.^18−22^ Indeed, acoustic waves emitted from unintentional defects and sample edges in free-standing flake samples are observed in the literature.^14,23,24^ However, the characterization of acoustic waves fully considering the dispersion relation and multimode nature in a finite size system has been limited in such samples. Here we characterize the nanoplate waves in a well-designed nanofabricated ultrathin plate and compare them with theoretical simulations. We use a silicon thin plate of *d* = 230 nm thickness and fabricate a 200 nm hole at the center, which acts as a wave source [Figure 1(b)]. First, we theoretically simulate how the nanoplate waves are generated in the nanofabricated silicon plate, based on the finite-element method. Then we experimentally visualize the nanoplate waves emitted from the wave source through the bright-field movie recorded by the ultrafast transmission electron microscope. We further characterize the emitted nanoplate waves by using Fourier-transformation analysis, which reveals the *f*-*k* characteristics.

The ultrathin single crystalline silicon plate was prepared using the focused ion-beam method, following the usual transmission electron microscopy specimen preparation of NB5000 (Hitachi High-Tech). We picked up the sample using a microprobe and then attached it to the copper mesh by tungsten deposition [Figure 1(b)]. To avoid damage around the hole, we first made a 3–μm-thick sample and then fabricated a hole of 200 nm diameter. After this process, the thickness of the sample was thinned to 230 nm. The thickness of the sample (230 ± 20 nm) was experimentally evaluated using the log-ratio technique^25,26^ of electron energy loss spectroscopy.

The detailed setup of the ultrafast transmission electron microscopy is described elsewhere.^24^ Experiments were performed using a Tecnai Femto (Thermo Fisher Scientific) instrument with an acceleration voltage of 200 kV. A 40 μm carbon-coated LaB_6_ tip (Applied Physics Technologies) was used as the photocathode. A 150 μm condenser aperture was used for all the measurements. Images of the bright-field and diffraction patterns were recorded with an UltraScan 1000XP charge-coupled device camera (Gatan). The spatial resolutions for all measurements are better than 10 nm. As a photon source, we used a Yb:KGd(WO_4_)_2_ solid-state laser (PHAROS, Light Conversion). The generated laser beam was split into pump and probe beams by a polarized beam splitter. The fundamental 1030 nm pump beam with a pulse duration of 290 fs passed through a delay line and was used to excite the sample. The rest of the beam passed through two β-Ba_2_B_2_O_4_ crystals for fourth-harmonic generation (257 nm) and was focused on the photocathode in the microscope. The repetition rate of the measurement was set at 25 kHz. In all the experiments, we used a pump fluence of 1.6 mJ/cm^2^. Temporal resolution was set to 5 ps although the maximum temporal resolution of our system is about 2 ps.^24^

To obtain the frequency-high-pass-filtered images in Figure 3, we applied a 3 × 3 × 3 median filter and a 3 × 3 × 3 average filter to the bright-field movie with *x*, *y*, and *t* axes. Then, we applied a fifth-order high-pass Butterworth filter along the time axis. To avoid the phase shift, we applied the frequency filter twice (once forward and once backward). The same frequency filter is applied to the calculation result in Figure 2. Such frequency filter results in the artifact at *t* < 0 in Figure 2(b) since applying high-pass frequency filter is mathematically equivalent to convolving the time-dependent data with the kernel of the high-pass filter that have finite width in real time. We confirmed that all values in Figure 2(b) are zero at *t* < 0 before applying the high-pass filter.

**Figure 2:**
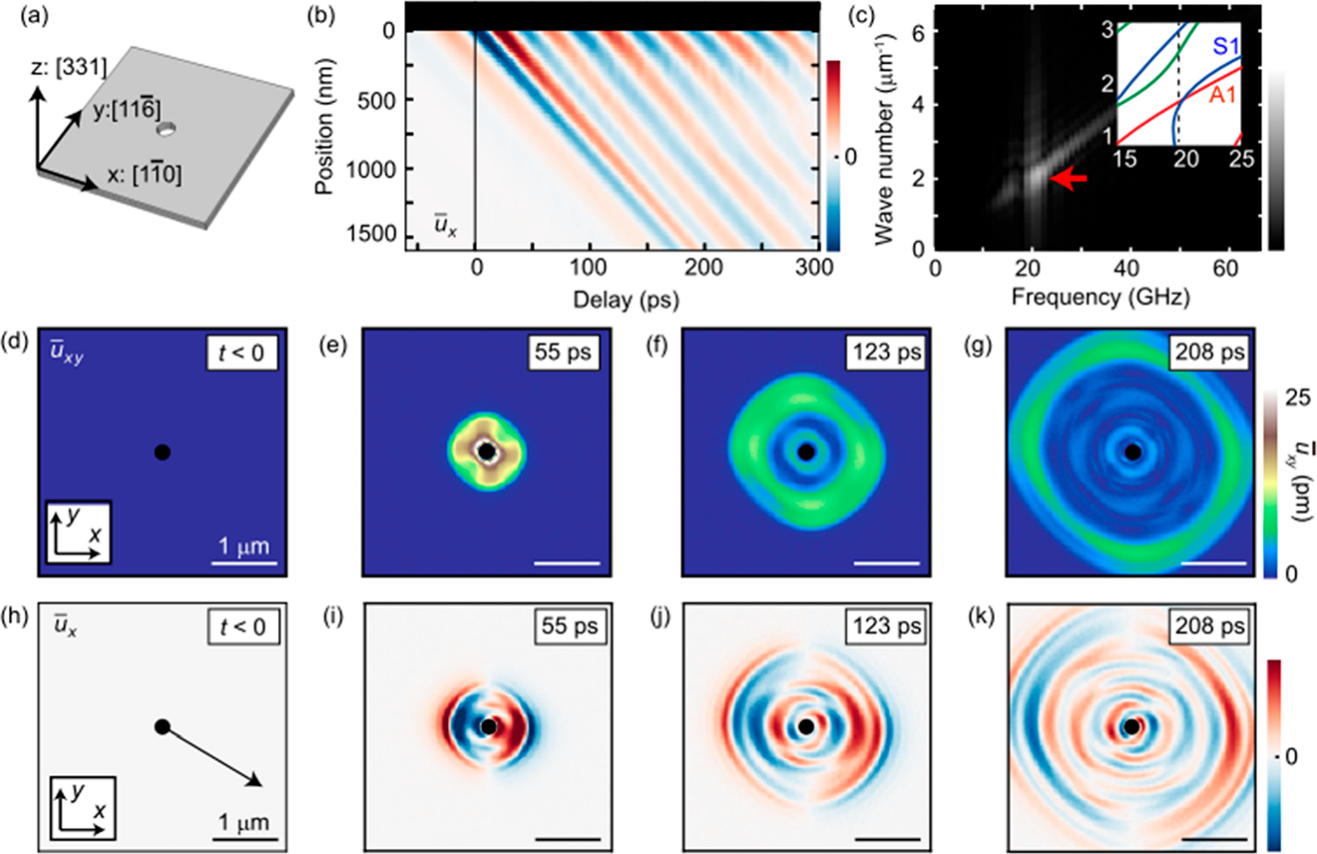
(a) Schematic of the nanofabricated silicon ultrathin plate used for the calculation (see Section 2 in the Supporting Information). (b) Space-time contour of frequency-filtered *u̅*_*x*_ along the black line in part h. The red and blue areas represent the anti-nodes of the periodic nanoplate waves. The finite *u*_*x*_ at *t* < 0 is an artifact due to the frequency filter. (c) Fourier-transformed data of part b. The red arrow indicates a peak at frequency *f* ≃ 20 GHz and wavenumber *k* ≃ 2 μm^–1^. The inset shows the dispersion curve along the black arrow in part h (the whole dispersion relation is shown in Figure S1). (d–g) Time dependence of *u*_*xy*_. (h–k) Time dependence of frequency-filtered *u*_*x*_. The red and blue areas represent anti-nodes of the nanoplate waves.

First, we theoretically investigated the nanoplate waves in an ultrathin silicon plate based on the finite-element method presented in the literature^10^ (see Section 2 in the Supporting Information for details), as shown in Figures 2(a–c). We prepared a three-dimensional thin plate of [331] oriented single crystalline silicon with a thickness *d* = 230 nm to compare with the experimental results. We define the *x*, *y*, and *z* directions as the [11̅0], [116̅], and [331] directions, respectively. At the center of the plate, there is a hole with a diameter of 200 nm, which acts as a source of nanoplate waves [Figure 2(a)]. As a result of the finite-element method calculation, we assess the time- and position-dependent atomic displacement *u*_*i*_ (*x,y,z,t*) (*i* = *x*,*y*,*z*). Figures 2(d–g) show the time dependence of the lateral atomic displacement  before and after photoexcitation. We averaged the atomic displacement along the *z* direction to compare the calculated and experimental results. At *t* = 0, the photoinduced stress generates an acoustic pulse that is emitted from the hole and then propagates radially, which is seen as the relative intense change in *u̅*_*xy*_. The amplitude of the acoustic pulse is anisotropic owing to the elastic anisotropy of the silicon single crystal,^27^ which in some cases appears as the phonon focusing effect.^28^ In addition, we also found a periodic nanoplate wave following the acoustic pulse. To extract the nature of the periodic nanoplate waves, we applied a frequency-high-pass filter with a cutoff frequency of 10 GHz to *u̅*_*x*_ = *d*^–1^ ∫_0_^*d*^*u*_*x*_*dz* [Figures 2(h–k)]; the blue and red regions correspond to the anti-nodes of the periodic nanoplate waves. From these images, the wavelength of the emitted periodic nanoplate waves appears to be on the sub-micrometer scale. We can quantitatively evaluate the periodic nanoplate wave from the space-time contour of *u̅*_*x*_ [Figure 2(b)], taken along the black arrow in Figure 2(h). This direction is selected to compare with the experimental results, as discussed later. Figure 2(b) clearly indicates that periodic nanoplate waves with a period of ∼50 ps and a wavelength of ∼500 nm are emitted from the hole.

The corresponding distribution of strains in the *f*-*k* space is obtained by Fourier-transforming the *u̅*_*x*_ data, as shown in Figure 2(c). Here we can confirm a strong intensity at around wavenumber *k* ≃ 2 μm^–1^ and frequency *f* ≃ 20 GHz (red arrow), which reflects the periodic nanoplate wave. We found that the frequency of the periodic nanoplate wave is consistent with that of the lowest-order longitudinal acoustic resonance mode *f* = *v*/2*d* ≃ 20 GHz (*v* = 9.17 nm/ps, *d* = 230 nm) and therefore mainly determined by the sample thickness. We confirm the corresponding periodic modulation of the out-of-plane atomic displacement *u*_*z*_ with the same frequency, which is shown in Section 2 in the Supporting Information. The inset in Figure 2(c) shows the calculated dispersion of nanoplate waves along the black arrow in Figure 2(h). (The whole *f*-*k* dispersion curves and atomic displacement fields are depicted in Figure S1 and are similar to those along the [11̅0] direction in Figure 1.) Considering that the sample is almost homogeneously photoexcited and that the resulting stress is thus symmetric with respect to the *z* axis, we assign the peak at (*f*, *k*) ≃ (20 GHz, 2 μm^–1^) as an S1 mode [Figure 1(d)] rather than an A1 mode. It is noted that our finite-element calculation suggests that the S0 mode at *k* ∼ 3 μm^–1^ should have much weaker amplitude than the S1 mode and it is hard to distinguish from the present condition (for detail, see Section 4 in the Supporting Information). In addition, we found the relatively weak contribution at the *f* > 20 GHz region that can be attributed to the acoustic pulse. The acoustic pulse should be the superposition of various plate wave modes, and therefore, the Fourier transformation should show a continuous contribution on the wide region of the dispersion curves. We confirmed that these contributions from the acoustic pulse disappear when we use only the periodic part of *u̅*_*x*_ for the Fourier-transformation analysis (see Section 5 in the Supporting Information).

Next, we experimentally characterize the photoinduced nanoplate wave in a nanofabricated silicon thin plate by ultrafast transmission electron microscopy with nm × ps resolution, as shown in Figures 3(a–c). Figure 3(a) shows the bright-field image of the nanofabricated silicon sample recorded at *t* < 0. The white circular area at the center is the hole of 200 nm diameter. The time dependence of the raw bright-field images after photoexcitation is shown in Supporting Movie 1. Figures 3(d–g) show the snapshots of the bright-field image that is high-pass-filtered with a cutoff frequency of 10 GHz along the *t* axis to clearly show the temporal change. The red and blue regions represent the anti-nodes of the periodic nanoplate waves. The time-dependent high-pass-filtered images are also shown in Supporting Movie 2. As discussed in previous studies,^15,29^ photogenerated nanoplate waves cause deformations of the crystal plane, and the intensities of the diffracted electrons are modified by a change in the diffraction condition. Correspondingly, nanoplate waves can be observed in transmitted electron intensities, i.e., the intensity of the bright-field images. In the present case, Figures 3(d–g) mainly reflect the atomic displacement along the *x* direction because the sample is unintentionally tilted (<2°) along the *x* direction from the [331] direction. It is also noted that the bright-field image intensities are strongly dependent on the slight (<0.1°) bending and tilting of the thin sample before photoexcitation, which can be confirmed by the complex bright-field image intensities in Figure 3(a). We found that the nanoplate waves were emitted from the hole and propagated along the black arrow in Figure 3(d). Figure 3(b) depicts the space-time contour taken along the black arrow, to investigate the nature of the emitted nanoplate waves. Similar to the simulation results, the nanoplate waves are periodically emitted from the hole with a period of 50 ps. This period is consistent with the simulation and out-of-plane acoustic resonance frequency *f* = *v*/2*d* ≃ 20 GHz observed by ultrafast electron diffraction measurement^30−32^ (see Section 3 in the Supporting Information).

**Figure 3:**
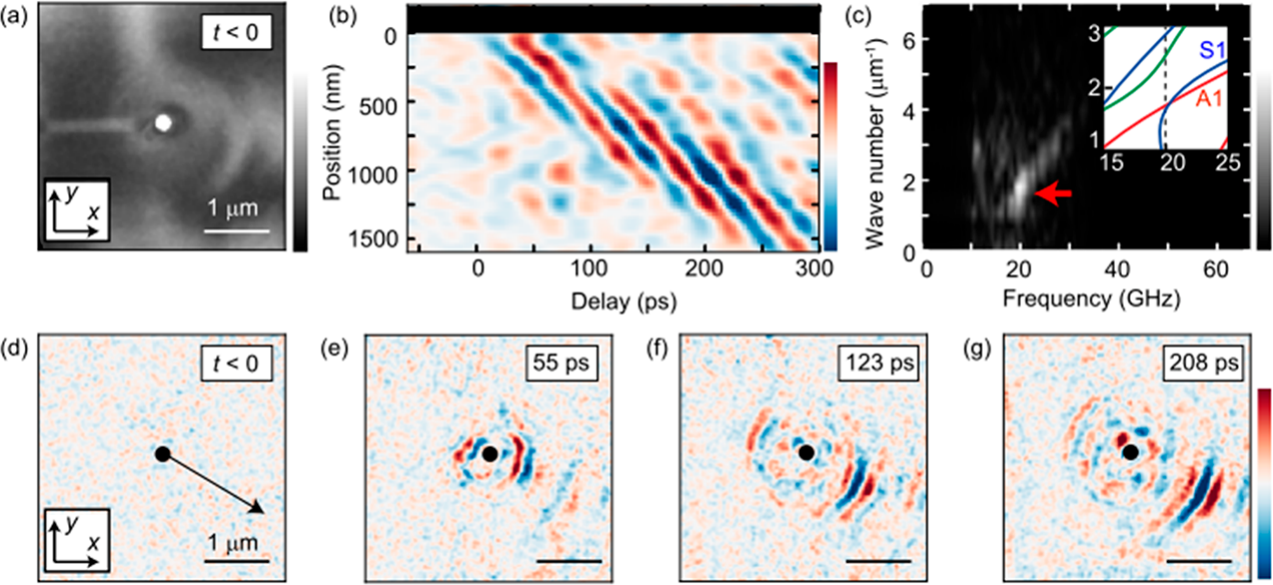
(a) Bright-field image of the nanofabricated silicon thin plate obtained before photoexcitation. The white region at the center is a hole of 200 nm diameter fabricated by the focused ion-beam method. (b) Frequency-filtered space-time contour of the bright-field image intensity along the black line in part d. The red and blue areas correspond to the anti-nodes of nanoplate waves. (c) Fourier-transformed data of part b. The red arrow indicates a strong contribution around frequency *f* ≃ 20 GHz and wavenumber *k ≃* 2 μm^–1^. The inset shows the calculated dispersion curve along the black arrow in part d (the whole dispersion relation is shown in Figure S1). (d–g) Frequency-filtered bright-field images at respective times.

We again applied Fourier transformation along both the horizontal and vertical axes of Figure 3(b) and obtained the distribution of the observed nanoplate waves in the *f*-*k* space in Figure 3(c). We observed a relatively weak contribution at the frequency range of >20 GHz, which may be attributed to the acoustic pulse predicted by the finite-element simulation. In addition, we also found a strong contribution at *f* ≃ 20 GHz and *k* ≃ 2 μm^–1^, which can be due to the periodic nanoplate wave. We also confirmed that the frequency of the periodic nanoplate wave is modified by changing the sample thickness (see Section 6 in the Supporting Information). The observed space-time contour in Figure 3(b) and its Fourier transformation in Figure 3(c) are well consistent with the theoretical simulations in Figures 2(b) and 2(c), indicating that the experimentally observed periodic nanoplate wave should correspond to the S1 mode. In the present case, the widths of the peaks at *f* ≃ 20 GHz and *k* ≃ 2 μm^–1^ do not reflect the intrinsic properties (e.g., lifetime) of the nanoplate wave but are rather limited by the range of the data acquisition. Such characterization will become also possible in a future work by taking a wide enough spatial and temporal measurement range (see Section 7 in the Supporting Information).

At present, it is difficult to estimate the photoinduced strain magnitude and the direction of atomic displacement only from the bright-field intensities, which are strongly affected by the local variation of strain due to the sample imperfectness. The theoretically expected change in *u*_*x*_ is in the order of picometers as shown in Figure 2(d–g), and therefore the present results suggest that even the very small (∼pm) atomic displacement can show up in bright-field movies through the periodic change in the diffraction condition induced by the sub-μm scale strain waves. We mention that further investigation of nanometric acoustic waves, including quantitative evaluation of amplitude and polarization, can be realized by ultrafast transmission electron microscopy when the recently developed 5-dimensional scanning transmission electron microscopy (5D-STEM) method^33,34^ is applied. Such quantitative mapping of the strain will enable more detailed discussion on the acoustic dynamics, such as the excitation mechanism of the nanoplate wave at the sample edge, in future studies.

The present results suggest a great potential of ultrafast transmission electron microscopy for the characterization of nanometric to submicron acoustic waves. Figure 3(c) clearly revealed the distribution of acoustic waves in *f*-*k* space, which can be directly compared with the dispersion calculation and finite-element simulation. Such investigation of the dispersion curve and distribution of acoustic waves by Fourier-transformation analysis has been applied to optical microscopy data in the sub-GHz × μm^–1^ range^17^ but not to ultrafast electron microscopy data. The previous electron microscopy measurements^14,15,24^ mainly focus on the sound velocities, and Fourier-transformation analysis has not been applied. This study shows that such Fourier-transformation analysis can be extended to better frequency and wavenumber regions of ultrafast electron microscopy, where the finite-size effect of nanostructure plays an important role as demonstrated in this study. Various nanostructures such as plates, wires, nanodots and semiconductor superlattices have a potential to host a variety of acoustic modes with nanometer wavelength. Direct observation of such acoustic modes by ultrafast transmission electron microscopy and Fourier-transform analysis is a suitable method for the characterization of acoustic waves in space, time, wavenumber, and frequency space with ps × nm resolution.

In conclusion, we demonstrated the experimental characterization of nanoplate waves in well-designed nanofabricated ultrathin silicon plates on the nm × ps scale. Our ultrafast transmission electron microscopy measurement clearly visualized the propagation of the photoinduced acoustic pulse and periodic nanoplate waves with sub-μm × ps spaciotemporal scales. Fourier-transformation analysis revealed the distribution of photoinduced strains in frequency-wavenumber space, which reveals that the periodic nanoplate wave is mainly composed of the S1 mode. These experimental results suggest that ultrafast transmission electron microscopy is a suitable method to characterize nm × GHz acoustic modes in various nanostructures.

## Supporting Information Available

Additional experimental and simulation data, as well as descriptions of the experimental and analysis methods, and finite-element calculations (PDF)
Time dependence of raw bright-field images after photoexcitation (MP4)
Time-dependent high-pass-filtered images (MP4)
