# Supplementary material for: Characterizing an Optically Induced Sub-micrometer Gigahertz Acoustic Wave in a Silicon Thin Plate
Source: Nano Lett. 2023 Mar 21;23(7):2490–5. doi: 10.1021/acs.nanolett.2c03938 (PMC10103304; doi:10.1021/acs.nanolett.2c03938)
Supplement: Supplementary file 1 — nl2c03938_si_001.pdf [file nl2c03938_si_001.pdf]

Supporting Information:

Characterizing an Optically Induced Sub-micrometer Gigahertz Acoustic Wave in a Silicon Thin Plate

Asuka Nakamura[1], Takahiro Shimojima[1], Kyoko Ishizaka[1,2]*

[1]RIKEN Center for Emergent Matter Science, Wako, Saitama 351-0198, Japan.

[2]Quantum-Phase Electronics Center and Department of Applied Physics, The University of Tokyo, Hongo, Tokyo 113-8656, Japan.

Section 1: Calculation of nano-plate wave dispersion

We calculated the dispersion curve and atomic displacement field in Figs. 1c, 1d, 2c, and 3c and Fig. S1 by using the partial wave technique described in the literature [2] under traction-free conditions at the surface. We assumed the two-dimensional sample of a flat plate form with 230 nm thickness along thickness direction (Z), and infinite length along the propagation direction (X). We also assumed that the sample is homogeneous along Y direction ($Y \perp X, Z$). We used the elastic constants of single crystalline silicon in the literature [35,36]. The whole dispersion curves of Fig. 2c and 3c are shown in Fig. S1.

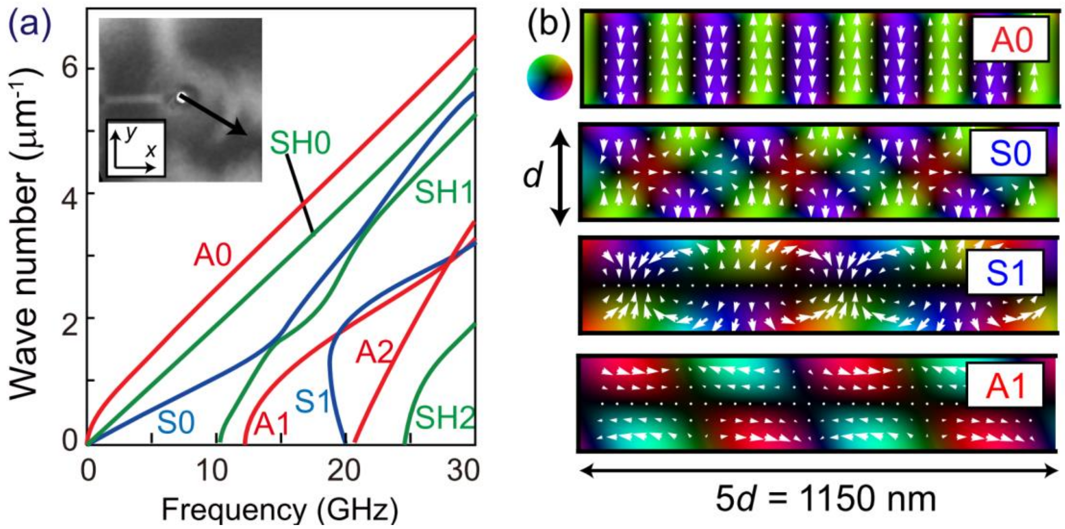

Figure S1 (a) Dispersion relation of a single crystalline silicon thin plate with a thickness of 230 nm along the black arrow in the inset. S0–S2 (A0–A2) represent symmetric (asymmetric) plate waves, which show symmetric (asymmetric) atomic displacement along the thickness direction, as shown in b. (b) Atomic displacement of A0, S0, S1, and A1 plate waves at 20 GHz.

Section 2: Finite-element simulation

For the finite-element method calculation, we extended the photo-induced elasto-dynamic equations in the literature [10] to a three-dimensional nanofabricated single crystalline system. We replaced Eqs (1) and (2) in Ref. [10] by a Christoffel equation

$$\rho\frac{\partial^2 u_i}{\partial \mathrm{t}^2} = \sum_{j,k,l=x,y,z} C_{ijkl}\frac{\partial u_k}{\partial j\partial l} + \nabla\sigma_{\mathrm{ext}},$$

where $\boldsymbol{u} = (u_x, u_y, u_z)$ is atomic displacements, ρ is density, C_{ijkl} is elastic constant, and σ_{ext} is a photoinduced stress term. We used the elastic constant of single crystalline silicon reported in Ref. [35,36]. The expression of σ_{ext} and the equations for the lattice temperature and carrier density were not changed from Ref. [10]. We assumed that the 1.2 eV pump light homogeneously excited the thin silicon plate since the optical penetration depth (approximately 1000 nm) was considerably larger than the sample thickness. Therefore, we set the source term $P(t)$ in Eqs. (3) and (4) in Ref. [10] to

$$P(t) = \frac{F/d}{\sqrt{2\pi}\Delta t}\exp\left(-\frac{t^2}{2\Delta t^2}\right).$$

We used a fluence F of 1.6 mJ/cm^2 and a pump pulse duration of Δt = 290 fs. The lateral length of the plate was set to be sufficiently large (8 μm) to avoid the acoustic wave emitted from the edge. The sample thickness was set to 230 nm. Other parameters and calculation conditions used for the simulation are presented in Ref. [10].

Section 3: Out-of-plane acoustic resonance mode

We confirm the periodic modulation of the out-of-plane atomic displacement $u_z$with 20 GHz frequency by the finite-element simulation. Fig. S2(b) shows time dependence of u_z at the red point in Fig. S2(a), where the nano-plate waves do not affect the u_z dynamics until 300 ps. u_z oscillates with a period of 50 ps. Fourier-transformed data in Fig. S2(c) shows sharp peak at approximately 20 GHz, which is consistent with the out-of-plane longitudinal acoustic resonance frequency v / 2d under the present sound velocity (v = 9.17 nm/ps) and the sample thickness (d = 230 nm). In addition, the third-order acoustic resonance mode with a frequency of 60 GHz is additionally seen in Fig. S2(c).

Such out-of-plane longitudinal acoustic resonance mode has been observed as a change in diffraction intensities as discussed in the previous ultrafast electron diffraction measurements [30–32]. Fig. S3(a) shows time dependence of the $\bar{9}$ 9 1 diffraction intensity. The oscillation with a period of about 50 ps is observed, as it is emphasized in the high-pass-filtered data in Fig. S3(b). The Fourier-transformation data in Fig. S3(c) again shows peak around 22 GHz, which is almost consistent with Fig. S2(c). There is a slight difference between the theoretically predicted (20 GHz) and experimentally observed (22 GHz) frequency of the out-of-plane acoustic resonance mode. This is due to the thickness variation of the sample. Indeed, our estimation of the sample thickness by EELS log-ratio technique shows about 10% variation

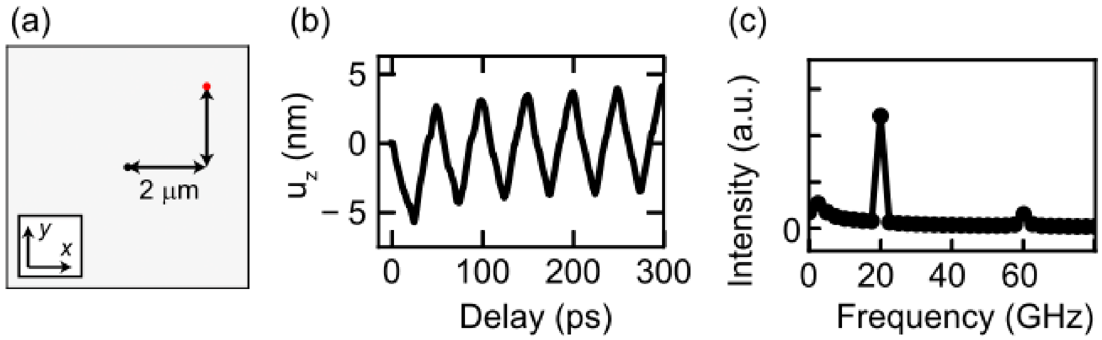

Figure S2 Out-of-plane standing wave calculated by finite-element simulation (a) Geometry of calculated silicon thin plate. The central black dot denotes a hole. u_z at the red point (x = y = 2 μm) is shown in b. (b) Time dependent u_z at the red point in a, where the nano-plate waves do not affect the u_z dynamics until 300 ps. (c) Fourier-transformed data of b. There is a sharp peak at approximately 20 GHz.

depending on the position of the sample. These experimental results strongly indicate the generation of the out-of-plane acoustic resonance mode in the present single crystalline silicon sample. Finally, we mention that the third-order acoustic resonance mode at 60 GHz cannot be distinguished in the present experimental data, possibly due to low signal-to-noise ratio although it is predicted by the simulation.

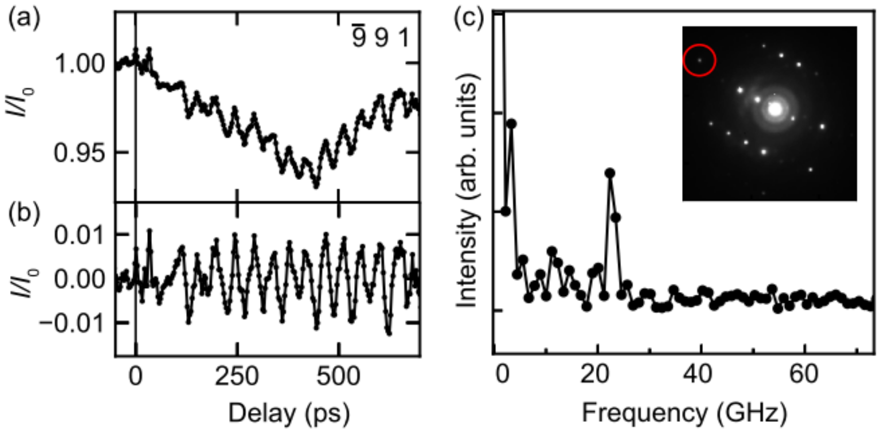

Figure S3 Out-of-plane standing wave revealed by an ultrafast electron diffraction measurement. (a) Time evolution of diffraction intensity I for $\bar{9}$ 9 1 diffraction, indicated by the red circle in c. The vertical axis is normalized by the diffraction intensity before photoexcitation I_0. (b) High-pass-filtered I/I_0 curve with the cutoff frequency of 10 GHz. (c) Fourier-transformed data of a. The inset shows the diffraction image of the silicon thin plate. The Miller indices of $\bar{9}$ 9 1 are not perpendicular to the surface normal [3 3 1], and thus, $I(t)$ in Fig. S3(a) can detect out-of-plane atomic displacements.

Section 4: S0 mode at 20 GHz

In this section, we describe the generation of S0 mode at 20 GHz frequency, which is not seen in the *f*-*k* distribution of nano-plate waves in Figs. 2(c) and 3(c). Figure S4(b) shows the time-dependent $\bar{u}_x$ along the whole arrow depicted in Fig. S4(a), obtained from the identical finite element calculation in the main text. By applying the Fourier-transformation to this data, we obtain the *f*-*k* distribution as shown in Fig. S4(c). Here we find that both S1 ($k \simeq 2$ μm^{-1}) and S0 ($k \simeq 3$ μm^{-1}) modes are photoexcited, although the amplitude of the S1 mode is much larger than S0. Such contribution from S0 mode is hard to distinguish in the simulated *f*-*k* image presented in the main text [Fig. 2(c)]. This is because the simulation data used for Fig. 2(c) is of narrower range (i.e. corresponding to the experimental setup), as shown in Fig. S4(d,e). Indeed, the contribution from the S0 mode at $(f, k) \simeq$ (20 GHz, 3 μm^{-1}) in Fig. S4(f) [= Fig. 2(c)] is strongly suppressed because of the insufficient spatial range. These indicate that the contribution from the S0 mode is hard to detect by experiment, even if it is photoexcited in reality.

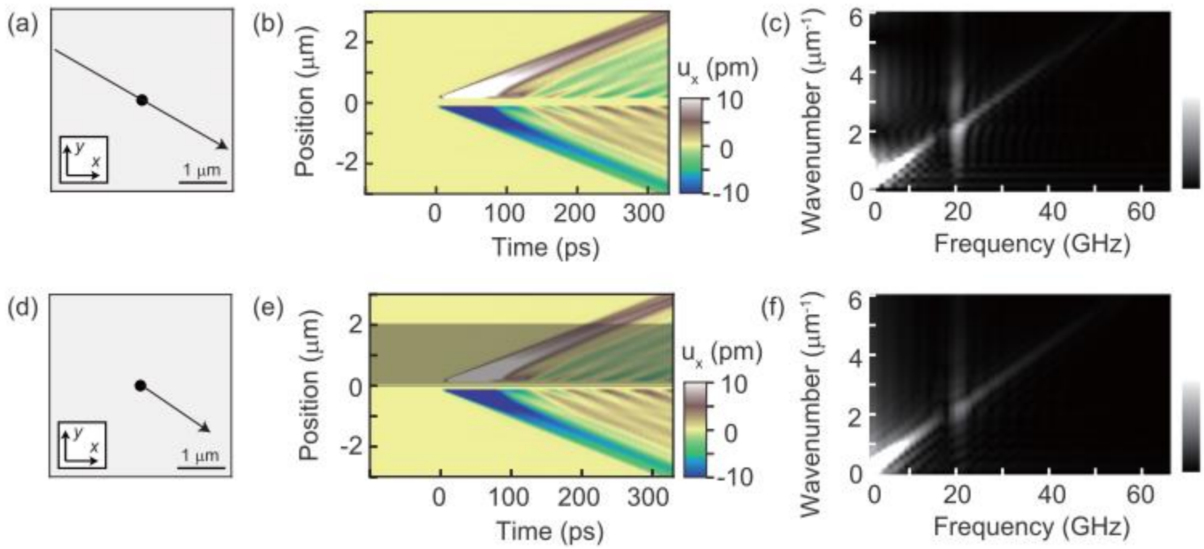

Figure S4 (a,d) Schematic of the simulation geometry. The black arrows indicate the region that is used for the Fourier-transformation analysis in c,f. (b,e) Time dependence of $\bar{u}_x$ along the black line in a,d. The gray shaded region is corresponding to the black arrow in d. (c,f) The Fourier transformation of b and gray shaded region in e, respectively.

Section 5: Frequency-wavenumber characteristics of the acoustic pulse

Fig. S5(a) shows the non-filtered $\bar{u}_x$ obtained from the identical finite element simulation in the main text, which also shows the opposite side of the central hole. In addition to the periodic oscillation with a period of 50 ps, there is an acoustic pulse emitted from the central hole at around $t = 0$, which shows relatively large amplitude as compared to the periodic plate waves. As discussed later, this acoustic pulse is the origin of the S1 modes extending above 20 GHz up to 40 GHz. When we apply the Fourier transformation along both spatial and temporal axes, all the dynamical contributions including acoustic pulse and periodic nano-plate waves are transformed together into the frequency-wavenumber space as described in the main text. Fig. S5(b) emphasizes the S1 mode extending above the resonance frequency up to ~60 GHz, by changing the color scale. On the other hand, when we use only the periodic part of $\bar{u}_x$ as shown in Fig. S5(c) for the Fourier transformation, we obtain Fig. S5(d). Although the frequency and wavenumber resolutions are severely limited due to the narrow spatial and temporal range of Fourier transformation, we can still distinguish the contributions at 20 GHz and 60 GHz (very weak), which correspond to the fundamental and 3rd-order acoustic resonance frequencies. On

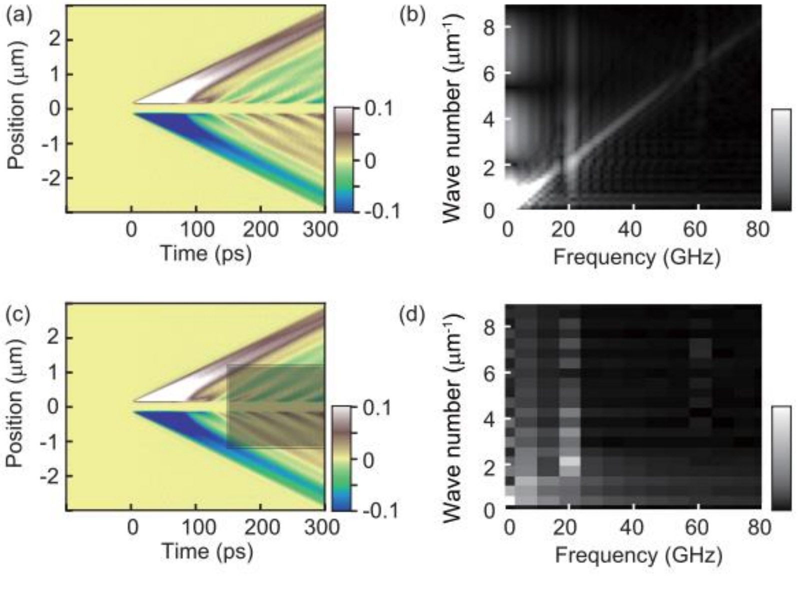

Figure S5: (a,c) Time dependence of $\bar{u}_x$, which is used in the main text. The gray shaded region in c is used to obtain the Fourier-transformation data in d. (b,d) The Fourier-transformed data of a and the gray shaded region in c, respectively.

the other hand, the extended continuous contribution between 20 and 60 GHz is strongly suppressed as compared to Fig. S5(b). Therefore the contribution between 20 GHz to 60GHz should be due to the pulse-like component which is generated only at the first impact of the optical-pulse irradiation.

Section 6: Thickness dependence of the photoinduced nano-plate wave

To clearly demonstrate the relation between the plate wave frequency and the plate thickness, we prepared a 450-nm thick Si single crystalline plate in the similar procedure with the 230-nm thick sample [Figs. S6(a,b)]. As revealed in the main text, the time dependence of bright-field image intensity of 230-nm sample in Fig. S6(c) show oscillation with a period of about 50 ps. Correspondingly, we found the peak at 20 GHz in Fourier-transformed data [Fig. S6(e)]. On the other hand, in the 450-nm sample, we observed ~100 ps oscillation as shown in Fig. S6(d), which is also confirmed as the 10 GHz component in the Fourier-transformed data in Fig. S6(f). These results well demonstrate that the frequency of the plate wave is proportional to the inverse of the sample thickness *d*.

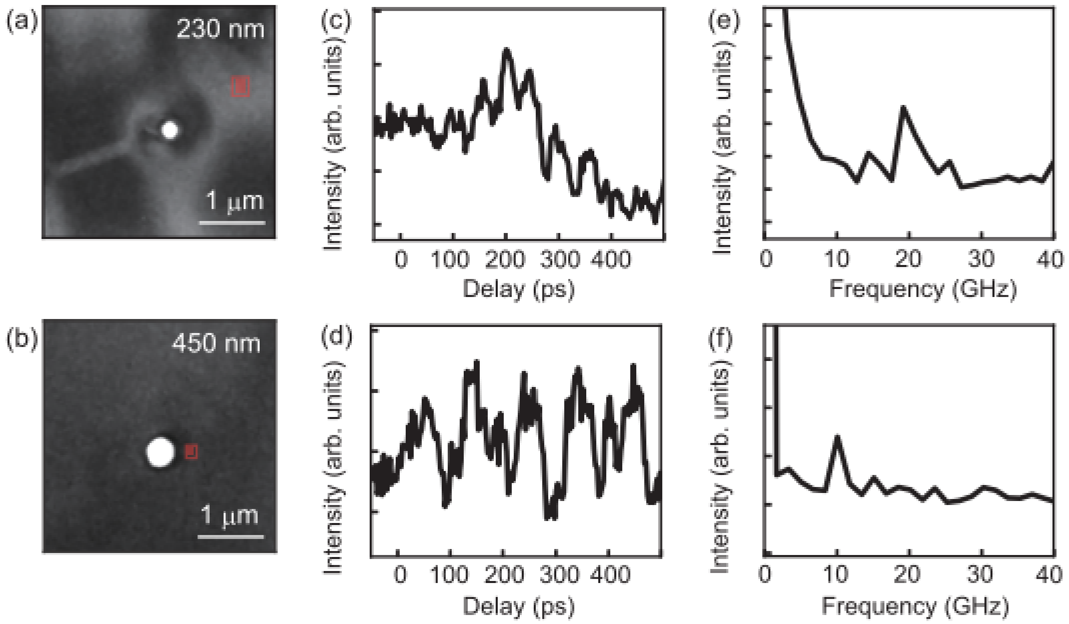

Figure S6: (a,b) Bright-field images of Si single crystal with thicknesses of 230 and 450 nm, respectively. (c,d) Time dependence of bright-field image intensities at red rectangles in a,b. (e,f) Fourier transformation of c and d, respectively.

Section 7: Width of the S1 mode in frequency-wavenumber space

To evaluate the “width" in experimentally obtained f-k data, we cut the image in Fig. S7(b) around k ~ 2 μm^{-1} and ω ~ 20 GHz as shown in Figs. S7(c,d). The gray shaded areas in Figs. S7(c,d) demonstrate the obtained “widths”. To elucidate the origin of these “widths”, we also show the Fourier-transformed image [Fig. S7(f)] of a reference data [Fig. S7(e)] that has the same range and number-of-points with the experiment, by using a most simple function

$$g(x,t) = \theta(t - c^{-1}x)\sin(2\pi ft - 2\pi kx),$$

where θ is Heaviside step function, the sound velocity $c = f/k$. We used f = 20 GHz and k = 1.9 μm^{-1}, the S1 mode at 20 GHz obtained by the dispersion curve calculation (see Sec. 1 in Supporting Information). The Heaviside step function $\theta(t - c^{-1}x)$ describes the simple propagation of the wave front, which also exists in the experimental data. As shown in Figs. S7(g,h), the Fourier transformation of this simple reference data already shows the similar widths along both frequency and wavenumber, reflecting the insufficient spatial and temporal ranges as

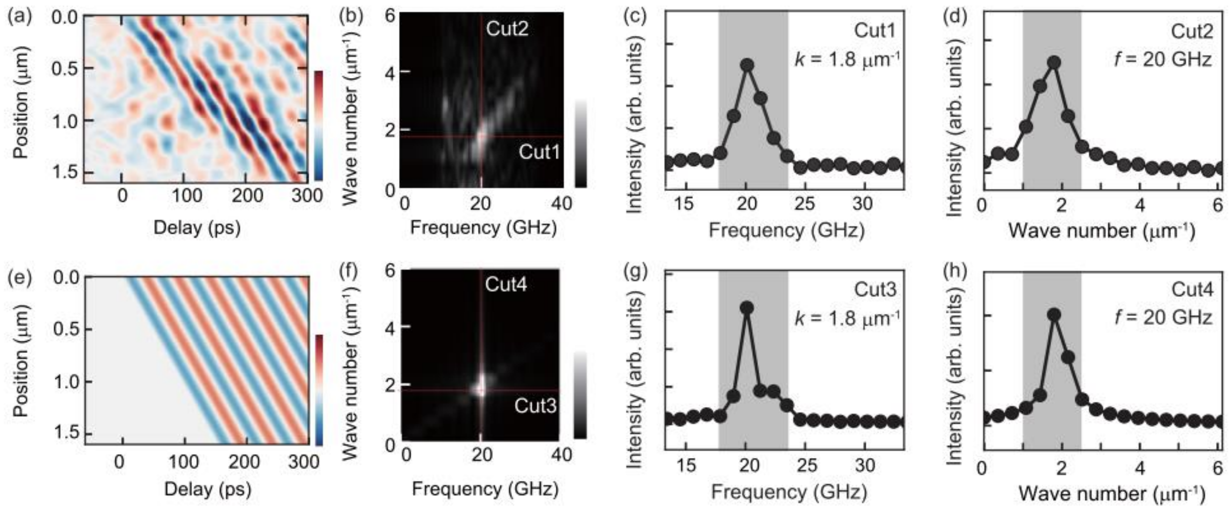

Figure S7: (a) The space-time contour of bright-field image [= Fig. 3(b)]. (b) The distribution of nano-plate waves in f-k space [= Fig. 3(c)]. (c,d) The line profiles along Cut1 and Cut2. (e) A reference data $g(x,t)$ that has the same range and number-of-points with the experiment. (f) The Fourier transformation of e. (g,h) The line profiles of f along Cut3 and Cut4.

compared to the wave-length and oscillation-period. Therefore, the present widths of frequency and wavenumber obtained from the experimental data in Figs. S7(c,d) can also be described by the same mechanism, and therefore does not reflect the nature of the nano-plate wave e.g. lifetime. Such discussion will also be possible in the future work when the spatial and temporal measurement range is sufficient.

References:

[35] H. J. McSkimin, W. L. Bond, E. Buehler, and G. K. Teal, *Measurement of the Elastic Constants of Silicon Single Crystals and Their Thermal Coefficients*, Physical Review **83**, 1080 (1951).

[36] J. J. Hall, *Electronic Effects in the Elastic Constants of N-Type Silicon*, Physical Review **161**, 756 (1967).
